# The Potential of Lamiaceae Herbs for Mitigation of Overweight, Obesity, and Fatty Liver: Studies and Perspectives

**DOI:** 10.3390/molecules27155043

**Published:** 2022-08-08

**Authors:** Farah Diab, Hawraa Zbeeb, Francesca Baldini, Piero Portincasa, Mohamad Khalil, Laura Vergani

**Affiliations:** 1 Department of Earth, Environment and Life Sciences (DISTAV), University of Genova, Corso Europa 26, 16132 Genoa, Italy; 2 Nanoscopy and NIC@IIT, Istituto Italiano di Tecnologia, 16152 Genoa, Italy; 3 Clinica Medica “A. Murri”, Department of Biomedical Sciences and Human Oncology, Medical School, University of Bari “Aldo Moro”, 70124 Bari, Italy

**Keywords:** Lamiaceae, medicinal plants, phytochemicals, nutraceutics, obesity, non-alcoholic fatty liver disease (NAFLD)

## Abstract

Numerous plants, plant extracts, and plant-derived compounds are being explored for their beneficial effects against overweight and liver diseases. Obesity is associated with the increased prevalence of non-alcoholic fatty liver disease (NAFLD), becoming the most common liver disease in Western countries. Obesity and NAFLD are closely associated with many other metabolic alternations such as insulin resistance, diabetes mellitus, and cardiovascular diseases. Many herbs of the Lamiaceae family are widely employed as food and spices in the Mediterranean area, but also in folk medicine, and their use for the management of metabolic disorders is well documented. Hereby, we summarized the scientific results of the medicinal and nutraceutical potential of plants from the Lamiaceae family for prevention and mitigation of overweight and fatty liver. The evidence indicates that Lamiaceae plants may be a cost-effective source of nutraceuticals and/or phytochemicals to be used in the management of metabolic-related conditions such as obesity and NAFLD. PubMed, Google Scholar, Scopus, and SciFinder were accessed to collect data on traditional medicinal plants, compounds derived from plants, their reported anti-obesity mechanisms, and therapeutic targets.

## 1. Introduction

Medicinal plants have a long history in traditional medicine for the management of diseases. Lamiaceae is the largest family of the Lamiales order, including more than 7000 species, mostly shrubs and herbs. Lamiaceae are typically cultivated as ornamental plants, but many of them are used as food and spices [1]. Many bioactive molecules have been isolated from Lamiaceae including alkaloids, phenolic acids, phenyl propanoids, flavonoids, terpenoids and other compounds such as hydrocarbons, sugars, lignans, and lignins. Plant parts (leaves, stems, or roots), extracts, and/or isolated compounds from Lamiaceae have been largely investigated for their pleiotropic biological effects, mainly antimicrobial, anti-inflammatory, antioxidant, and cytotoxic activities. A large body of studies has suggested the potential of the Lamiaceae in ameliorating metabolic disorders, those related to obesity in particular. Most of these studies focused on the pharmacological potential of the Origanum species for their antidiabetic [2], antihyperlipidemic [3], anti-obesity [4], anti-inflammatory [5], and antioxidant properties [6]. However, *Ocimum* spp. [7], *Salvia* spp. [8], and *Mentha* spp. [9] have also been studied for treating metabolic disorders. A recent study has investigated the antisteatotic and antioxidant activities of *Thymbra spicata* L. extract using in vitro models of non-alcoholic fatty liver disease (NAFLD) [10]. The present review is an effort to summarize the traditional uses, phytochemistry applications, and nutraceutical potential of the most cited species of Lamiaceae in treating obesity and its complications.

### Obesity and Non-Alcoholic Fatty Liver Disease

The World Health Organization (WHO) began sounding the alarm on global obesity in the 1990s and introduced the term “epidemic” in reference to obesity. Obesity is a condition characterized by the excessive accumulation of fat in the body. It is not an outcome of a single disorder, but a combination of genetic, metabolic, food habits, environmental, physical activity, and social factors. Obesity is often associated with a significantly higher risk of developing insulin resistance, type 2 diabetes mellitus (T2DM), metabolic syndrome, NAFLD, atherosclerosis, cardiometabolic dysfunction, and cancer [11]. In 2016, more than 1.9 billion adults worldwide were overweight, and over 650 million out of these were obese, with the worldwide prevalence of obesity having nearly tripled between 1975 and 2016. In 2020, about 39 million children under the age of 5 were overweight or obese.

NAFLD, the major cause of liver diseases in Western countries, is defined as an excessive fat accumulation, particularly triglycerides (TGs), in the liver (≥5% in liver parenchyma), in the absence of other liver disorders [12]. Obesity, especially central obesity, is the main causative factor of excessive fat accumulation in the liver. The clinic-pathologic spectrum of NAFLD ranges from simple steatosis to non-alcoholic steatohepatitis (NASH), which can progress to fibrosis, cirrhosis, and hepatocellular carcinoma [13]. NAFLD is a multisystem disease that could affect multiple organs and regulatory pathways, resulting in an increase in the risk of several chronic diseases [14]. Recently, the “double-hit” hypothesis has been replaced by the “multiple-hit” hypothesis, offering a more comprehensive delineation of the pathogenesis of NAFLD [15].

To date, no ideal pharmacological treatment is available for NAFLD [16]. Therefore, lifestyle modification, which includes a healthy diet and vigorous physical activity along with weight reduction, remains the first line treatment for NAFLD. However, due to the poor adherence to this type of treatment, especially for long-term weight loss diets, some of which may have harmful effects on the liver, there is significant interest in identifying therapeutic agents for the treatment and/or prevention of NAFLD progression. The potential adverse effects of conventional medical therapies led to identify novel complementary therapies that are both natural and safe products, such as herbal medicine and functional foods (e.g., fruits, vegetables), dry materials or their extracts [17].

## 2. Methodology

### 2.1. Data Extraction

The selection of the plants included in our study among the large family of Lamiaceae was solely based on the number of published studies regarding their efficacy in treating obesity-related disorders. For that purpose, a comprehensive search of published studies was conducted between June 2021 and July 2022 using different electronic databases. PubMed, Google Scholar, Scopus, SpringerLink, ScienceDirect, and SciFinder were accessed to collect data on traditional medicinal plants, compounds-derived from these plants, their reported anti-obesity mechanisms, and therapeutic targets. The taxonomically accepted name of each plant in this review has been vetted from “The Plant List” (available online: www.theplantlist.org) or MPNS (http://mpns.kew.org) (accessed on 20 July 2021).

Pre-set search strings like “Lamiaceae” with “obesity”, “overweight”, “diabetes”, “hyperlipidemia, “hypercholesterolemia”, “NAFLD”, “antioxidant”, “inflammation”, “hepatoprotection” and “pharmacology”, combined with the Boolean operators AND/OR were used to select plants.

### 2.2. Studies Selection Process and Distribution among Authors

After selection of the Lamiaceae plants to be included in the study, each author focused on a group of these and, independently, read the selected full articles. Each writer followed the same process that consisted of three steps. In the first stage, articles based on the selected keywords were searched. In the second stage, the titles and abstracts of all the resources were screened. The third step consisted in another screening of the selected titles and abstracts to confirm whether the content fit the research aims. It should be noted that only articles published in English were included. Review articles, conference abstracts, case reports and protocol papers were excluded from the selection process. The data from each full article from all the authors was presented to ensure its consistency, and disagreements among the authors were resolved by discussion.

### 2.3. Result Report

In total, after discarding the overlapping references, 97 papers including in vitro, in vivo, and clinical trials out of 250 were selected in our review to display the most important and relevant studies.

## 3. Lamiaceae: General Aspects of the Family

Lamiaceae is a plant family belonging to the Lamiales order, and it consists of 236 genera and 7200 species [18]. The family was known for a long time as Labiatae (nomen conservandum) or the mint family, but in the 1820 the name was changed to Lamiaceae [18]. Lamiaceae includes the highest number of aromatic herbs, shrubs, and trees with quadrangular branches. The most important genera of this family are Salvia, Scutellaria, Stachys, Plectranthus, Hyptis, Teucrium, Vitex, Thymus, and Nepeta [19].

**Figure 1:**
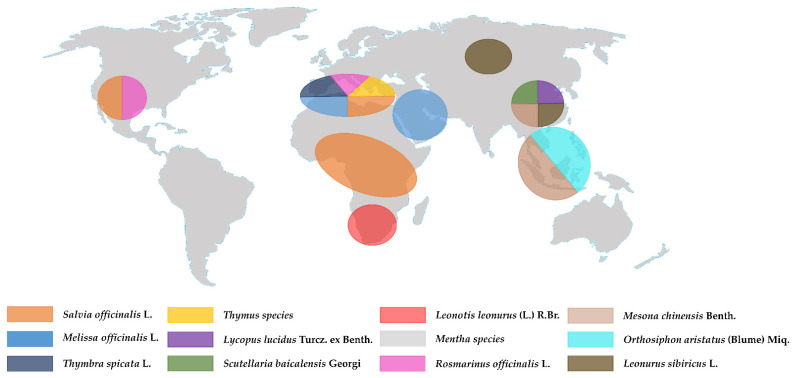
Geographical representation of the worldwide distribution of different Lamiaceae plants.

**Figure 2:**
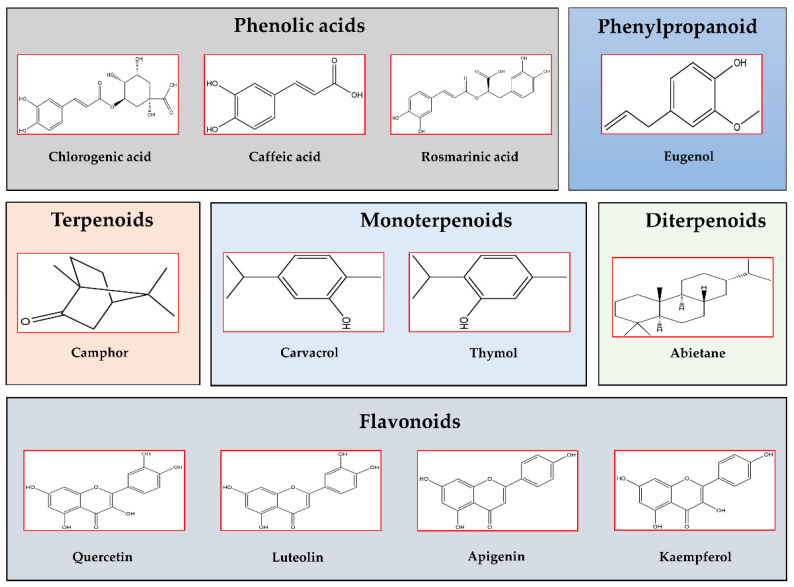
Chemical structures of the most abundant and common bioactive compounds found in Lamiaceae plant species (ChemDraw2020 software, PerkinElmer Informatics, Waltham, MA, USA). Phenolic acids: Chlorogenic acid, Caffeic acid, Rosmarinic acid; Phenylpropanoid: Eugenol; Terpenoids: Camphor; Monoterpenoids: Carvacrol, Thymol; Diterpenoids: Abietane; and Flavonoids: Quercetin, Luteolin, Apigenin, Kaempferol.

Lamiaceae inhabit different ecosystems and are widely cultivated [20], but it has been noticed that the major distribution of Lamiaceae species is in the Mediterranean area, as shown in Figure 1. Most of the species are aromatic and contain a mixture of bioactive compounds which led to their use in the cosmetic, pharmaceutical, and food industries, mainly under form of essential oils [21]. Figure 2 reports the different classes of the common phytochemical constituents of Lamiaceae plant species such as phenolic acids, phenylpropanoid, monoterpenoids, diterpenoids, terpenoids, and flavonoids. Moreover, Lamiaceae species are promising potential sources of natural antioxidants, owing to their high polyphenol content. Natural antioxidants from various botanical sources have been widely investigated as a potential alternative to synthetic antioxidants; for this reason, plants of Lamiaceae are regarded as a source of functional foods. We wish to emphasize this as the WHO suggests that plants with a long history of applications in folk medicine should be thoroughly evaluated for possible use in the treatment and prevention of diseases, and the Lamiaceae perfectly fall into this field. Table 1 summarizes the most abundant bioactive compounds found in each species, the corresponding applications, and the number of citations for each.

**Figure 3:**
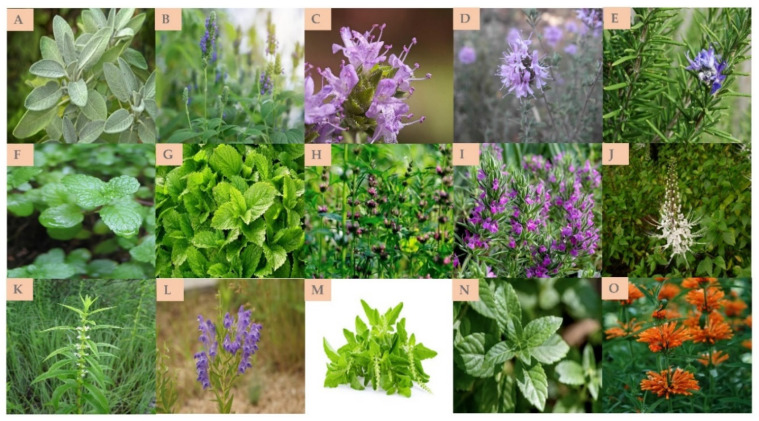
A Group of Lamiaceae plants (Available online: www.theplantlist.org, accessed on 5 August 2022) included in the review. (**A**): *Salvia officinalis* L., (**B**): *Salvia hispanica* L., (**C**): *Thymbra capitata* (L.) Cav., (**D**): *Thymus saturejoides* Coss., (**E**): *Rosmarinus officinalis* L., (**F**): *Mentha spicata* L., (**G**): *Melissa officinalis* L., (**H**): *Leonurus sibiricus* L., (**I**): *Thymbra spicata* L., (**J**): *Orthosiphon aristatus* (Blume) Miq., (**K**): *Lycopus lucidus* Turcz. ex Benth., (**L**): *Scutellaria baicalensis* Georgi., (**M**): *Ocimum species*, (**N**): *Mesona chinensis* Benth., (**O**): *Leonotis leonurus* (L.) R.Br.

In the following chapter, we summarize the scientific results regarding the most cited plants of the Lamiaceae family that have been investigated for their efficacy in treating and/or preventing obesity and obesity-related diseases through in vitro and in vivo studies (animal models) (Table 2), up to clinical trials (Table 3). The corresponding picture for each plant species is well-documented in Figure 3.

## 4. Lamiaceae: Bioactive Properties

### 4.1. Salvia Species

*Salvia* is the largest genus of the Lamiaceae, as it includes roughly 900 aromatic species of herbaceous, perennial, biennial, and annual plants [22]. This genus covers almost all continents including the Pacific Islands, Central Asia, the Mediterranean, tropical Africa, and America [22]. Common species include *Salvia officinalis* L. (common sage), *Salvia hispanica* L. (chia), *Salvia miltiorrhiza* Bunge (Chinese sage), and *Salvia sclarea* L. (clary sage), with the first two being the most studied ones.

*Salvia officinalis* L. is a perennial subshrub native to the Mediterranean area, but certainly naturalized in Southern France and Spain, widely used in both culinary and medicinal preparations. Its name “sage” originates from the old Latin word “salvarem” meaning ‘to heal’, and in fact *S. officinalis* is rich in bioactive compounds, mostly diterpenoids (abietane and labdane) and phenolic compounds (caffeic acid derivatives). The long history of sage as medicinal plant was confirmed by studies showing its promising potential in ameliorating heart diseases and obesity [23]. Sage was shown to also be effective in improving inflammation [24], hyperlipidemia, hypercholesterolemia, and T2DM [25,26,27]. Table 2 depicts the numerous studies on the in vitro and in vivo beneficial effects of *Salvia* spp. against obesity and obesity-related disorders. In streptozotocin-induced diabetic rats, the alcoholic extract of sage leaves decreased blood glucose (GLU), TG, total cholesterol (Chol), cyclooxygenase-2 (COX2), urea, uric acid, creatinine, aspartate transaminase (AST), alanine transaminase (ALT) and increased insulin secretion [25], while the methanolic extract decreased blood GLU and insulin levels, and improved the lipid profile and the insulin sensitivity by reducing the plasmatic pro-inflammatory cytokines: tumor necrosis factor alpha (TNFα), interleukin-12 (IL-12), and increasing the anti-inflammatory cytokines: IL-2, IL-4, and IL-10 [28]. A study on male Wistar rats demonstrated the effects of sage leaves in regenerating/restoring Langerhans islets to their normal size [27]. Moreover, sage as a food supplement seems to act in the prevention of T2DM by inhibiting hepatic gluconeogenesis and/or glycogenolysis [29]. The oral administration of sage to diabetic rats up-regulated the expression of insulin and insulin-regulated glucose transporter (Glut-4), and inhibited α-glucosidase activity. Moreover, the sage leaves inhibited lipogenesis in adipocytes by reducing the accumulation of lipid droplets [28].

An in vivo study on ovariectomized rats revealed that sage leaves possess estrogenic activity that led to loss in the body weight and restored the plasma lipid levels to the normal profile by reducing total Chol, high-density lipoprotein (HDL-Chol), low-density lipoprotein (LDL-Chol), TG, total lipids, very-low-density lipoprotein (VLDL) [30] In humans, a double-blind clinical trial was carried out on 80 diabetic patients being divided into two groups which received *S. officinalis* tablets (150 mg extract) or placebo, three times a day for 90 days; both the plasma GLU and Chol levels significantly decreased in *S. officinalis* treated patients [31]. Another study demonstrated the efficacy and safety of incapsulated *S. officinalis* leaf ethanolic extract on 86 hyperlipidemic diabetic patients treated with placebo or 500 mg capsule every 8 h by the oral route for three months. An improvement of the glycemic and lipid profile with a reduction in the fasting GLU, glycated haemoglobin (HbA1c), total Chol, TG, and LDL-Chol and elevating HDL-Chol was observed [32]. A pilot trial was carried out on six female volunteers to investigate the effects of sage being consumed as tea and showed that sage tea improved the lipid profile, antioxidant defences, and lymphocyte 70kDa heat shock proteins (Hsp70) expression [26] (Table 3).

*Salvia hispanica L.* is an annual herbaceous plant, originally from Mexico and Guatemala, but recently has also been cultivated in Australia, Bolivia, Columbia, Peru, Argentina, America, and Europe [33]. It is commonly known as chia, a name that originates from the Spanish word “chian” meaning oily. Indeed, *S. hispanica* is extremely rich in ω-3/-6 fatty acids [33], α-linolenic acid in particular, which are precursors of prostaglandins, leukotrienes, and thromboxanes. Chia seeds are an ancient food that is tracked back to the pre-Columbian era, when they were also used in religion [34]. *S. hispanica* provides a balanced amount of nutrients composed of insoluble fibers, proteins with a high quality of amino acids, high content of antioxidants, such as phenolic compounds such as caffeic acid, chlorogenic acid, and quercetin. Many studies described its potential for treating obesity [35], diabetes [35,36,37], hypertension [36], cardiovascular disease [37], NAFLD [38], hyperlipidemia [39], inflammation and oxidative stress [40,41]. 

**Table 1:** An overview summary on the most abundant bioactive compounds in each species, its applications, and the number of citations for each.

List of Plants	Most Abundant Bioactive Compounds	Applications
*Salvia officinalis* L.	Diterpenoids: abietane and labdane Phenolic compounds: caffeic acid derivatives	Folk medicine
*Salvia hispanica* L.	Caffeic acid, chlorogenic acid, and quercetin	Uses in food, folk medicine, primary cosmetics, and a part of religious rituals
*Thymus species*	Thymol, carvacrol apigenin, luteolin, thymusin, rosmarinic, and caffeic acid and derivatives	Traditional phytomedicine, food, food additive, spicy, and herbal tea
*Rosmarinus officinalis* L.	Carnosic acid, rosmarinic acid, camphor, caffeic acid, ursolic acid, betulinic acid, and carnosol	Traditional phytomedicine, food additives, and herbal tea
*Mentha species*	Menthol, luteolin, rosmarinic acid, Kaempferol, and hesperidin	Traditional phytomedicine, food, food additive, spicy, herbal tea
*Melissa officinalis* L.	Rosmarinic acid, geranial, neral, luteolin, naringin, hesperidin, and caffeic acid and derivatives	Traditional phytomedicine, food flavoring, and herbal tea
*Leonurus sibiricus* L.	Chlorogenic acid, caffeic acid, and quercetin	Herbal medicine
*Thymbra spicata* L.	Carvacrol and rosmarinic acid	Culinary ingredient: in salad and tea infusion Herbal medicine
*Orthosiphon aristatus* (Blume) Miq.	Rosmarinic acid	Folk medicine
*Lycopus lucidus* Turcz. ex Benth	Rosmarinic acid and derivatives Flavonoid: chrysoeriol, luteolin, quercetin, isoquercitrin, and rutin	Traditional phytomedicine
*Scutellaria baicalensis* Georgi	Flavonoid: Baicalein, wogonoside, and wogonin	Traditional phytomedicine
*Ocimum species*	Eugenol, rosmarinic acid, apigenin, luteolin, β-sitosterol, and carnosic acid	Traditional phytomedicine, food additive, spicy, and fragrance agent
*Mesona chinensis* Benth.	Caffeic acid	Traditional phytomedicine, gelatin-type dessert, and herbal beverage
*Leonotis leonurus* (L.) R.Br.	Marrubin and premarrubin	Traditional phytomedicine

The in vivo study on male Wistar rats [39] investigated the anti-steatotic effect of chia seeds by taking four rat groups fed DE (diet of standard food) and 4 groups fed DC (diet with added chia) for 4 weeks. After this period the groups were further divided into 2 control groups, two groups received tyloxapol to induce acute dyslipidemia, 2 groups received (CCl_4_) to induce acute steatohepatitis, and the last 2 groups were treated with tyloxapol + CCl_4_ to induce acute dyslipidemia along with NASH. The results showed that chia intake, partially or totally prevented cholestasis, liver damage, inflammation, oxidative stress, and markedly lowered the TG and Chol in both dyslipidemic and NASH groups compared to controls. It is possible that the hypolipidemic and hepatoprotective effects of chia may be correlated to its high content of α-linolenic acid. On the other hand, the chia intake improved antioxidant defence by increasing superoxide dismutase (SOD) expression and activity, peroxisome proliferator-activated receptor (PPARα) expression, catalase (CAT) activity, and HDL-Chol levels, and decreased the concentrations of total Chol, LDL-Chol, and the inflammatory markers IL-1β, as well as the lipid profile and liver indices [40]. Chia seed supplementation for rats fed a sucrose-rich diet reduced the abdominal and thoracic circumferences, carcass fat content, adipose tissue weights, and visceral adiposity index, and this was accompanied by an improvement in insulin sensitivity and plasma lipid profile through a reduction in both FAT/CD 36 plasma membrane and the fat synthesis enzyme activities: ATP-citrate lyase, FAS cell surface death protein (FAS), G6PD, and PEPCK [42]. The supplementation of chia seeds to Wistar rats fed a sucrose-rich diet improved both the activities of the antioxidant enzymes CAT, SOD, and glutathione peroxidase (GPx), as well as the mRNA expression of (Nrf2), and led to a decrease in xanthine oxidase (XO) activity and reactive oxygen species (ROS) contents, and in the plasma pro-inflammatory cytokines IL-6 and TNFα [43]. Moreover, chia supplementation increased the PPARγ expression and the ω-3/-6 fatty acid ratio of membranes, suggesting that chia seed may improve the dysfunction induced by a sucrose excess, and reduced adipocyte hypertrophy by improving lipogenic enzyme activities, and decreased lipid storage, GLU phosphorylation and oxidation in skeletal muscle [44]. Moreover, chia seeds seem to play a cardioprotective activity in normalizing the systolic blood pressure [45] (Table 2). In a clinical trial a single arm experimental study investigated the effects of the daily intake (25 g/day) of chia seeds by 25 patients with NAFLD, showing a decrease in body weight, total Chol, non-HDL-Chol, and circulating free fatty acids (FFA) [38]. On the other hand, a double-blind, randomized, controlled trial with two parallel groups of 77 obese patients with T2DM [35] indicated the beneficial potential of chia seeds in promoting weight loss, and improving obesity-related risk factors such as a decrease in c-reactive protein (CRP) and an increase in plasma adiponectin. A single-blind study of over 20 subjects with T2DM evaluated the effect of the daily intake (37 ± 4 g/day) of chia seeds for 12 weeks and found that chia attenuated the a major cardiovascular risk factor [37]. In the same context, the daily consumption of 40 g of chia seeds over 12 weeks is more than enough to reduce the systolic blood pressure of diabetic patients [36] (Table 3).

### 4.2. Thymus Species

The genus *Thymus* L. contains about 300 species of aromatic perennial herbaceous plants and subshrubs that are native to temperate regions in Eurasia, but now are cultivated throughout the world.

*Thymus vulgaris* L. (common thyme) is the most common herb of this genus. It is native to Italy and the western Mediterranean, where it is widely used as food and herbal medicine. It is slightly spicier than oregano and sweeter than sage. Fresh thyme is rich in rosmarinic acid and luteolin, but thymol is the most abundant phenolic compound present in thyme essential oil [46]. Many in vitro and in vivo studies reported the antioxidant and anti-inflammatory effects of *T. vulgaris*, where it was able to decrease a wide range of inflammatory mediators such as ROS, reactive nitrogen species (RNS), and pro-inflammatory cytokines [47]. As illustrated in Table 2, in a mouse model of liver damage, *T. vulgaris* played hepatoprotective effects by decreasing oxidative stress and inflammatory markers [48], and in gentamicin-treated rats, it had hypolipidemic and anti-inflammatory effects by normalizing the plasmatic AST and ALT and bilirubin level, as by restoring the normal lipid parameters and ROS production [49].

*T. saturejoides* Coss. is a Moroccan perennial shrub locally known as “Azkouni” or “Zaitra”. It is largely employed in traditional medicine to treat hypertension, T2DM, cold, fever, dermatological and circulatory disorders [50]. In streptozotocin-treated rats (model of T2DM), *T. saturejoides* was reported to reduce blood GLU and body weight and improve GLU tolerance [51].

Other Thymus species seem to have anti-diabetic potential: *T. schimperi* Ronniger seems to lower the blood GLU in alloxan-insulted diabetic mice [52], and *T. praecox* Opiz restores GLU homeostasis, ameliorates insulin resistance, and improves pancreatic β-cell function in streptozotocin/nicotinamide-induced diabetic rats [53].

In humans, a randomized, controlled, double-blind, crossover human trial was carried out on hypercholesterolemic patients to investigate the effect of virgin olive oil enriched with thyme phenolic compounds (PC). The daily dietary intake for three weeks of a PC-enriched virgin olive oil decreased blood oxidized LDL [54], and improves the expression of chol efflux-related genes [55]. This cardioprotective effect could be mediated by the modulation of gut microbiota by the increase in populations of bifidobacteria together with increases in PC microbial metabolites with antioxidant activities (Table 3).

### 4.3. Rosmarinus officinalis L.

*R. officinalis* L. (Rosemary) is native to countries in southern Europe such as Portugal, as well as Asian Mediterranean Countries (Lebanon, Syria, and Palestine) [56], and was largely used as a food and natural medicine for over a million of years. *R. officinalis* possesses many different biological properties that are mainly due to the presence of volatile and phenolic compounds [57]. Extraction of bioactive compounds from rosemary showed that rosmarinic, carnosol, and carnosic acids are the most abundant compounds [57]. A lot of in vitro studies reported the ability of *R. officinalis* extracts to exert antioxidant and antidiabetic activities (Table 2). Its leaves extract decreased the level of ROS in hydrogen peroxide (H2O2)-insulted Hela cells [58], stimulated the glycolysis and fatty acid oxidation through activating AMP-activated protein kinase (AMPK) and PPAR pathways in cultured human hepatocellular carcinoma cells (HepG2) subjected to hypoglycemic conditions [59]. Moreover, leave extract was able to modulate human adipocyte differentiation and to interfere with adipogenesis and lipid metabolism [60], to improve the insulin resistance in fatty acid-loaded L6 myotubes through stimulation of GLU uptake and AMPK phosphorylation, and to decrease the activation of c-Jun *N*-terminal kinases, mammalian targets of rapamycin and 70-kDa ribosomal protein S6 kinase [61].

**Table 2:** Main in vitro and in vivo (animal models) studies with the different species of the Lamiaceae family.

Plants	Models		Treated Disorders	Proposed Mechanisms	Ref.
*Salvia officinalis* L.	In vivo	Male Wistar rats	Diabetes Hypoglycemia	↑Insulin secretion ↓Serum GLU, TG, TC, urea, uric acid, creatinine, AST, and ALT	[25]
	In vitro In vivo	3T3-L1 pre-adipocyte cell line HFD-fed mice (C57Bl6)	Diabetes Hyperlipidemia Obesity	↓Blood GLU, TNF-α, KC/GRO, and IL-12 ↑ IL-2, IL-4, and IL-10 Improvement in HOMA-IR, TG, and NEFA ↓Body weight and LDs	[28]
	In vivo	Male Wistar rats	Diabetes Hyperlipidemia Obesity	Improvement in serum creatinine and UA concentrations ↓α-amylase and lipase activities ↓Serum AST, ALT, and LDH ↓Body weight	[27]
	In vivo	Female Balb/c mice and male Wistar rats	Diabetes	↓Gluconeogenesis Inhibition of hepatic GLU production by glucagon	[29]
	In vivo	Female Wistar rats	Hyperlipidemia Obesity	↓plasma Chol, HDL-Chol, LDL-Chol, TG, total lipids, and VLDL ↓Body weight	[30]
*Salvia hispanica* L.	In vivo	Male Wistar rats	Dyslipidemia NAFLD/NASH	Prevention of cholestasis elevation (AP, GGTP, and TB) ↓ALT ↓Liver and plasma TNF-α ↓TG and total Chol ↓LP and CAT activities	[39]
	In vivo	Adult female Wistar rats	Hyperlipidemia	↑SOD and CAT activities ↑PPAR-α expression ↑HDL-Chol ↓TC, IL-1β, VLDL-Chol, & LDL-Chol ↓NFκB expression	[40]
	In vivo	Male Wistar rats	Obesity Dyslipidemia	Improvements in insulin sensitivity and plasma lipid profile (TG, FFA, & Chol) ↓FAT/CD 36 plasma membrane levels ↓ Fat synthesis enzyme activities (ATP CL, FAS, G-6-P DH, and PEPCK) ↓PKCβ and SREBP-1 protein levels	[42]
	In vivo	Wistar rats SRD-fed	Dyslipidemia Insulin resistance	↓Body weight ↑CAT, SOD, & GPx activities ↑SOD and GPx mRNA ↑ PPAR-α protein level ↑Nrf2 expression ↑n-3/n-6 FA ratio of membrane phospholipid ↓IL-6 and TNF-α	[43]
	In vivo	Wistar rats SRD-fed	Dyslipidemia Insulin resistance	↓Adipocyte hypertrophy, cell volume, and size distribution ↓Lipogenic enzyme activities (ACC, FAS, ME, and G-6-PDH) ↑Hexokinase and PDHc activities ↑GLUT-4 protein mass ↑Glycogen storage, G-6-P concentration, and GSa activity	[44]
	In vivo	Wistar rats SRD-fed	Dyslipidemia Insulin resistance	↓Systolic blood pressure ↑GIR ↓Lipid storage (TG, LC ACoA, and DAG) and plasma FAs ↑PDHa ↑FAT/CD36 protein mass level ↓M-CPT1 and PPARα activity	[45]
*Thymus vulgaris* L.	In vivo	Sodium nitrite-treated mice	Liver damage	↓ AST, ALT, MDA, IL-1β, IL-6, TNF-α, ↑ GSH and SOD activities	[48]
	In vivo	Gentamicin-treated rats	Liver damage	↓ AST, ALT, bilirubin, total lipids, ROS	[49]
*Thymus saturejoides* Coss.	In vivo	Streptozotocin-treated rats	T2DM	↓Blood GLU and weight Improve GLU tolerance	[51]
*Thymus schimperi* Ronniger	In vivo	Alloxan-induced Diabetic Mice	Diabetes	↓Fasting blood GLU	[52]
*Thymus praecox* Opiz	In vivo	Streptozotocin/nicotinamide-induced type 2 diabetic rats	Diabetes	↓Blood GLU ↑α-glucosidase, PEPCK, GLUT-2 and SGLTs	[53]
*Rosmarinus officinalis* L.	In vitro	Hela cells	Oxidative stress	↓ROS	[58]
	In vitro	HepG2 cells	Obesity	↑AMPK, ACC, LDLR and PPARα ↓G6Pase	[59]
	In vitro	Preadipocytes	Obesity	↓TG ↓ CDK4, CCND1 and CDKN1A ↑ GATA3 and WNT3A	[60]
	In vitro	L6 myotubes	Insulin resistance	Restored insulin-simulated GLU uptake ↓ palmitate induced phosphorylation in IRS-1 ↑AMPK ↓ JNK and mTOR	[61]
	In vivo	Male Wistar rats	Oxidative stress	↓TBARS, H2O2 ↑ GSH ↑SOD, CAT, GPx and GST activities	[62]
	In vivo	Rats	Liver toxicity	↑SOD, CAT and GPx activities ↓ MDA ↓neutrophils and macrophages ↓ hepatocytes necrosis and fibrosis	[63]
	In vivo	Rats	Hypercholesterolemia	↓Chol, HDL and TBARS ↑SOD, CAT and GPx activities	[64]
	In vivo	Mice	Inflammation	↓ COX2, PGE-2, IL- 1b, MMP2 and NO	[65]
*Mentha spicata* L.	In vivo	Nicotine-induced liver damage in Wistar rats	Liver damage	↓AST, ALP, ALT, LDH and MDA	[66]
*Mentha pipertia* L.	In vivo	Rats	Liver damage	↓ALT, AST, ALP, and LDH ↓Lipid peroxidation ↓gamma glutamyl transferase, urea and creatinine	[67]
	In vivo	Rats	Liver damage	↓p53 Improve TGF-β1 expression	[68]
	In vivo	Rats	Liver damage	↓ALT, AST, ALP, and LDH ↓Lipid peroxidation ↓gamma glutamyl transferase, urea and creatinine	[67]
*Mentha villosa* Huds	In vivo	HFD-fed mice	NAFLD Obesity	↓blood GLU, insulin, leptin and TG ↑ adiponectin ↓ IL-6, TNF-α and SEBP 1c ↑ AMPK	[69]
*Melissa officinalis* L.	In vitro	HUVECs	Oxidative stress	↑ cell viability ↓ [hydroperoxide]	[70]
	In vitro In vivo	HUVECs mice	Obesity	↓ body weight gain, adipose tissue mass and adipocyte size ↓VEGF-A, FGF-2 and MMPs mRNAs	[71]
	In vitro In vivo	HepG2 cells mice	Obesity	↓ body weight gain ↓ visceral fat mass ↓ adipocyte size ↓ hepatic lipid accumulation ↑ expression of PPARα target genes	[72]
	In vitro In vivo	HepG2 cells mice	NASH	↑ SOD, CAT and GPx activities ↑ AMPK, PPARα and CPT-1L ↓ α-SMA and COL1A1	[73]
	In vivo	Mice	Oxidative stress	↓Mn-induced TBARS levels ↑ SOD, CAT, GPx	[74]
	In vivo	Rats	Diabetes	↓ weight, hyper-glycemia, hypo-insulinemia and hepatic lipid accumulation ↑ AMPKα2, ACOX, MCAD, VLCAD ↓ IL-6 and CD68 restored β-cell mass	[75]
*Leonurus sibiricus* L.	In vitro	INS-1E cells	Diabetes	↑ Insulin secretion ↑ Insulinoma cell proliferation	[76]
	In vivo	C57BL/6 mice	Hypercholesterolemia	↓ Plasma cholesterol ↑HDL-Chol ↑SOD, CAT, GR & GPx activities ↓TBARS and protein carbonyls	[77]
	In vivo	C57BL/6 mice	Obesity	↓ Serum TG, TC, and LDL-Chol ↑ HSL and ATGL expression	[78]
*Thymbra spicata* L.	In vitro	Rat hepatocytes FaO cells Human endothelial HECV cells	Steatosis Endothelial dysfunction	↓ Hepatic lipid accumulation ↓ ROS and lipid peroxidation	[10]
	In vivo	HFD-fed mice	NAFLD	↓ TC, LDL-Chol, TG, and MDA ↑ HDL-Chol ↑GSH, SOD, and CAT activities	[79,80]
*Thymbra capitata* (L.) Cav.	In vivo	Paracetamol-insulted rats	Liver damage	↑ SOD and GPx	[81]
*Orthosiphon aristatus* (Blume) Miq.	In vivo	C57BL/6 mice	Hyperlipidemia Obesity	↓ Body weight, TG, TC, and LDL-Chol ↓ Hepatic LDs ↓ MDA ↑ SOD activity	[82]
	In vivo	Sprague Dawley rats	Diabetes	↑ GLP-1 and ghrelin levels	[83]
*Lycopus lucidus* Turcz. ex Benth	In vitro In vivo	HepG2 cells HFD-fed mice	NAFLD	↓Intracellular lipid accumulation ↓lipogenesis ↑ β-oxidation ↓body weight, relative liver weight, serum ALT, total Chol, LDL, serum GLU, insulin, leptin, and TNF-α ↓SREBP-1 ↑ PPAR-α	[84]
	In vitro	Human umbilical vein endothelial cells (HUVEC)	High glucose- induced Vascular inflammation	↓ cell adhesion molecules (CAMs) ↓ ROS production ↓ NFκB expression	[85]
	In vivo	Mice	Liver injury	↓ serum ALT, AST, ALP, TG, total Chol, and total bilirubin ↑ hepatic GSH contents ↑ SOD and CAT activities ↓ hepatic MDA ↓ DNA fragmentation	[86]
*Scutellaria baicalensis* Georgi	In vitro In vivo	HepG2 cells Mice	NAFLD	↓TG and Chol ↑AMPK	[87]
	In vivo	Type 2 diabetic db/db mice	Obesity	↓ weight gain, TG, ALT and hyperinsulinemia ↓p-AMPK	[88]
	In vivo	Mice	Insulin-resistance	↓Fasting and postprandial GLU, fasting insulin, HOMA-IR, TG and LDL-Chol ↓adipose tissue macrophages, CD11b^+^; Kupffer cells, TNF-α	[89]
*Ocimum gratissimum* L.	In vivo	Ovariectomized rats	Obesity	↓Body weight ↓Adipocyte size	[90]
*Ocimum tenuiflorum* L.	In vivo	Rats	Dyslipidaemia	↓Lipogenesis ↑Mitochondrial fatty acid uptake ↓Insulin resistance ↑GSH, GPx, CAT	[91]
	In vivo	Rats	Hyperlipidemia	↓Lipid accumulation ↓Oxidative stress	[92]
	In vivo	Rats	Diabetes	↓Glucose ↓TG ↓creatinine ↓ Carbohydrate metabolism enzymes	[93,94]
*Mesona chinensis* Benth.	In vitro In vivo	RAW 264.7 cells Male mice	Immune deficiency	↑SOD, CAT and GPx activities ↓MDA	[95]
*Leonotis leonurus* (L.) R.Br.	In vivo	Rats	Diabetes mellitus	inhibited fresh egg albumin-induced paw edema and hypoglycemic effects in rats	[96]
	In vitro In vivo	INS-1 cells Obese rats	Hyperglycemia	↑ GLUT2 expression ↑RR and MM potential ↑ insulin	[97]
	In vitro In vivo	3T3, Chang, C2C12, INS-1 Obese Wistar rats	Obesity	↑ PPAR ↑glucokinase ↑FAS and UCP2 ↓leptin	[98]

↓, significantly decreased; ↑, significantly increased.

Carnosic acid, a major component of leaves, is known for its antioxidant activity due to its ability as a 2,2-diphenyl-1-picrylhydrazyl (DPPH) radical scavenger [99]. It also shows anti-adipogenic activity as it decreases body weight, liver weight, blood and hepatic TG and total Chol levels, and through the activation of expression of lipolysis-related genes (CPT1) [100,101] and inhibiting α-Glucosidase enzyme [99]; however, its low solubility limits its applications [102].

The antioxidant and hepatoprotective potential of both leaves of essential oil [62] and leaves of ethanol extract [63] of *R. officinalis* were investigated in rats exposed to chromium and CCl_4_ to induce hepatotoxicity. The results demonstrated their ability in preventing oxidative damage and restoring the antioxidant enzymes levels to normal, decreasing lipid peroxidation and preventing hepatocytes necrosis and fibrosis. Moreover, the antidiabetic potential of different *R. officinalis* leaves extracts were summarized by decreased levels of total serum HbA1c, GLU, Chol and TG, and increased wound healing ability when they were investigated on different animal models with induced NAFLD [103], hyper Chol [64], T2DM [104], and obesity [105]. Lastly, the anti-inflammatory activity of *R. officinalis* was characterized by decreased levels of inflammatory biomarkers: COX2, prostaglandin E2, IL-1b, matrix metallopeptidase (MMP2), and nitric oxide (NO) when tested on carbon tetrachloride (CCI4)-induced rats [65]. In humans, a clinical study carried out on 40 adults (mean age 56 years) diagnosed with T2DM, the intake of rosemary tea (2 g/L of water per day) for 90 days was assessed in order to evaluate whether using rosemary tea instead of powder might have a similar therapeutic effect in the treatment of T2DM, since a large number of leaf powder capsules are required (10 capsules daily) [106]. The results indicate a decrease in mass index, waist-hip ratio, lipid peroxidation, insulin resistance, and the pancreatic β-cell function (Table 3). It was concluded that shortening time and dose, as well as changing the formulation of the *R. officinalis* constitutes a promising treatment for drug-resistant T2DM patients.

### 4.4. Mentha Species

The genus *Mentha* (Mint) grows worldwide, especially in South Africa, Australia and the temperate regions of Eurasia [107], and includes 38 species of aromatic, almost exclusively perennial plants. While members of Mentha are known as the “true mints”, some other genera of Lamiaceae use mint in their common name. Mentha plants are cultivated for the aromatic essential oil contained in the stems and leaves, which is used for culinary, cosmetic and medicinal purposes [108]. Mint oils are currently one of the most valuable essential oils worldwide. Spearmint (*Mentha spicata* L.) and peppermint (*Mentha piperita* L.) are the most important commercial species.

*Mentha spicata* L. is known for its anti-diabetic potential. Oral administration of an aqueous extract of *M. spicata* leaves significantly reduced blood GLU level in diabetic rats [109]. On the other hand, *M. spicata* seems to possess hepatoprotective activity. In rats with nicotine-induced liver damage, *M. spicata* administration significantly reduced AST, Alkaline phosphatase (ALP), ALT, lactate dehydrogenase (LDH), and lipid peroxidation in rat liver [66] (Table 2).

*Mentha piperita* L. is widely consumed as a food ingredient, essential oil, and tea infusion. *M. piperita* extracts and oil have been used in traditional medicine [110]. Studies on cellular and animal models demonstrated a wide range of biological and pharmacological functions. While rosmarinic acid is the main phenolic compound in the aqueous extract, menthol and menthone are more abundant in the essential oils of *M. piperita*. The essential oil showed beneficial effects on diabetic rats: treatment with 40 mg/kg alleviated hyperglycemia, improved the antioxidant defence and led to the regeneration of the liver and pancreas tissues [111]. The hepatoprotective ability of *M. piperita* leaf extract against arsenic-induced hepatotoxicity was observed in Swiss albino mice, leading to a reduction in acyl carrier protein, ALP, serum glutamate oxaloacetate transaminase (SGOT), serum glutamate pyruvate transaminase (SGPT), and lipid peroxidation levels [112]. Similarly, *M. piperita* essential oil exerted a protective effect against CCl_4_ -induced hepatotoxicity and renal failure in rats by reducing lipid peroxidation, ALT, AST, ALP, LDH, gamma glutamyl transferase, urea and creatinine, and enhanced antioxidant enzymes [67]. *M. piperita* essential oil also demonstrated antifibrogenic effects against CCl_4_-induced liver fibrosis by improving the antioxidant status, suppressing p53, and subsequently modulating transforming growth factor (TGF-β1) expression [68] (Table 2).

*Mentha villosa* Huds (the synonym of *Mentha cordifolia*) is another popular herb typically used both as flavour in Thai food and as herbal tea. It has been shown to improve lipid metabolism and glycemia in obese mice treated with *M. villosa* leaf extract for six weeks, leading to a reduction in GLU, insulin, leptin, TG levels, and in the inflammatory cytokines IL-6 and TNFα, and an increase in adiponectin level, accompanied with the activation of the AMPK signaling pathway [69] (Table 2).

*M. longifolia* (L.) L. is known for its potential in treating hypertension. In humans, a randomized, blind, placebo-controlled trial including 29 subjects with mild hypertension showed that the hydro-alcoholic extract of M. longifolia leaves led to a dose- and duration-dependent reductions in systolic and diastolic blood pressures as well as in mean arterial blood pressures, thus confirming an effective, safe and promising potential for this plant as a phyto-therapeutical for hypertension therapy [113] (Table 3).

### 4.5. Melissa officinalis L.

*M. officinalis* L., also known as lemon balm or honey balm, is a bushy herbaceous perennial herb that is typically grown in herb gardens and border fronts for its lemon-scented leaves. It is native to southern Europe and the Mediterranean region, West Asia, and North Africa [114]. Traditionally, it is consumed as an herbal tea to improve digestion, reduce gastrointestinal disorders, reduce sleep disturbances, and for its antispasmodic properties [115]. The bioactive components of *M. officinalis* are mainly found in the leaves; the essential oil is rich in volatile compounds, terpenoids (monoterpenes, sesquiterpenes, triterpenes), and polyphenolic compounds [rosmarinic acid, caffeic acid, protocatechuic acid, quercitrin, rhamnocitrin, luteolin]. For this reason, the use of *M. officinalis* essential oil is suggested for the prevention and management of diseases like hyperlipidemia, T2DM, and other metabolic syndromes [114].

**Table 3:** Main clinical controlled trials on the intake of Lamiaceae plant species.

Plants	Sample Size	Gender (Age)	Participants	Format, Dose	Duration of Study	Action	Ref.
*Salvia officinalis* L.	*n* = 80	Men and women	T2DM patients	tablets (150 mg extract/three times/day)	90 days	↓2hpp blood sugar and Chol	[31]
	*n* = 86	Men and women	Hyperlipidemic T2DM patients	extract capsules (500 mg/8 h)	90 days	↓GLU, HbA1c, total Chol, TG, and LDL-Chol ↑HDL-Chol	[32]
	*n* = 6	Women (40–50 years)	Diabetic patients	Tea, (300 mL/twice a day)	28 days	↓Plasma LDL-Chol and total Chol ↑HDL-Chol ↑Hsp70 expression ↑SOD and CAT activities	[26]
*Salvia hispanica* L.	*n* = 25	Men and women	NAFLD patients	Milled chia (25 g/day)	70 days	↓Body weight, total Chol, FFA, and non-HDL-Chol	[38]
	*n* = 77	Men and women (35–75 years)	Overweight and obese patients with T2DM	ground chia (30 g/1000 kcal daily)	180 days	Weight loss ↓CRP ↑Plasma adiponectin	[35]
	*n* = 20	Men and women (64 ± 8 years)	T2DM patients	37 ± 4 g/day	84 days	↓SBP ↓hs-CRP ↓ vWF	[37]
	*n* = 42	Men and women (21–65 years)	T2DM patients	Chia seeds (40 g/day)	84 days	↓SBP	[36]
*Thyme* spp.	*n* = 12	Men and women (46–67 years)	Hypercholesterolemic patients	25 mL/day	3 weeks	↓ox-LDL ↑bifidobacteria	[54]
	*n* = 22	Men and women (46–64 years)	Hypercholesterolemic patients	25 mL/day	3 weeks	↑expression of key cholesterol efflux regulators	[55]
*Rosmarinus officinalis* L.	*n* = 40	Men and women (mean age 56)	Type 2 diabetes	Tea, 2 g/L/day	90 days	↓ body mass index ↓ waist-hip ratio ↓ HbA1c ↓insulin resistance ↓ lipid peroxide levels	[106]
*Mentha longifolia* (L.) L.	*n* = 29	Men and women (40–65 years)	Blood hypertension patients	300 mL/day	16 weeks	↓Systolic (SBP), diastolic blood pressures (DBP), mean arterial blood pressures (MAP)	[113]
*Melissa officinalis* L.	*n* = 58	Men and women (25–65 years)	Hyperlipidemic patients	Two capsules (500 mg each) after meals, 3 times/day	2 months	↓ LDL	[114]
	*n* = 62	Men and women (20–65 years)	T2DM patients	two capsules (each 350 mg)/day	12 weeks	↓HbA1c, TG and hs-CRP ↑HDL	[116]
*Mesona chinensis* Benth.	*n* = 40	Men (20–40 years)	overweight	HC meal + 0.5 or 1 g of extract	4 h	↓MDA and serum TG ↑antioxidant status	[117]

↓, significantly decreased; ↑, significantly increased.

Many studies described the antioxidant and antidiabetic potentials of different *M. officinalis* extracts (Table 2). Both the radical scavenging activity [115] and the antioxidant potential in human endothelial cells (HUVECs)-insulted cells [70] were reported. In cellular models the *M. officinalis* extracts were able to decrease the expression of angiogenic factors, and of MMP-2 and MMP-9 in HUVEC cells [71], and to stimulate the expression of PPARα target genes acting in fatty acid β-oxidation and lipolysis [72] and AMPK phosphorylation [73] in HepG2 cells. In mice, *M. officinalis* extracts were able to reduce lipid peroxidation and total thiol levels in the brain of Mn-insulted mice [74]. Moreover, *M. officinalis* extracts played anti-diabetic and hepatoprotective effects in hyperlipidemic rats by lowering total Chol, total lipid, the serum level of ALT and AST and lipid peroxidation. In established animal models of T2DM, M. officinalis extracts increased the production of fatty acid-oxidizing enzymes (AMPKα2, ACOX, MCAD, and VLCAD) in the liver, and inhibited pancreatic inflammation by reducing the expression of inflammatory factors IL-6 and CD68 [75]. In NAFLD mice, *M. officinalis* extracts led to a reduction in hepatic fibrosis [73]. Furthermore, some clinical studies on diabetic and hyperlipidemic patients showed that the daily administration of *M. officinalis* capsules resulted in decreasing their LDL, TG, and HbA1c, as well as decreasing the inflammatory biomarker hs-CRP; however, further investigations are needed [114,116] (Table 3).

### 4.6. Leonurus sibiricus L.

*L. sibiricus* L., commonly called honeyweed or Siberian motherwort, is a ubiquitous aromatic herb native to Russia, Mongolia, and China [118]. It is largely used as a culinary ingredient and in folk medicine for treating T2DM [76], hypercholesterolemia and oxidative stress [77], and weight loss [78]. The medicinal properties of *L. sibiricus* are attributed mainly to the phenolic acids, iridoid and phenylpropanoid glycosides, flavonoids, alkaloids, and labdane diterpenoids [119].

An in vitro study reported that both aqueous and methanolic extracts from *L. sibiricus* aerial parts enhanced insulin secretion and insulinoma cell proliferation in rat insulinoma cells (INS-1E) through depolarization of the plasma membrane and an increase in the intracellular calcium concentration [76]. Additionally, an in vivo study using C57BL/6 mice showed reduced plasmatic chol levels, lipid peroxidation and protein carbonyls, and increased HDL-chol levels and activation of the hepatic antioxidant enzymes SOD, CAT, glutathione reductase, and GPx upon administration of the ethanolic extract of *L. sibiricus* [77]. On the other hand, the aqueous extract of *L. sibiricus* aerial parts exerted anti-adipogenic effects (inhibition of weight gain) in ovariectomized mice by decreasing the serum TG, total chol, and LDL-Chol levels and positively regulating the hormone-sensitive lipase and Adipose triglyceride lipase expression [78] (Table 2).

### 4.7. Thymbra Species

The genus *Thymbra* includes many thyme-like plants native to the Mediterranean region, mainly Lebanon, Turkey, and Greece [120]. *Thymbra spicata* L. (known in Lebanon as wild Za’atar) is traditionally used as a food and herbal tea, and in folk medicine as an antiseptic agent and to relieve headaches, toothaches, colds, asthma, and rheumatism [120], and this can be greatly attributed to its richness in phenolic compounds including phenolic acids (rosmarinic acid), phenolic monoterpenoids (carvacrol, thymol), and flavonoids (both glycosides and aglycones) [121].

The species *T. spicata* has gained much popularity as a remedy to combat hypercholesterolemia and oxidative stress [79]. In vivo studies conducted on high-fat diet (HFD) fed mice showed that both ethanolic and aqueous extracts of *T. spicata* aerial parts possess anti-hypercholesterolaemic, antioxidant, and anti-steatohepatitic potentials by reducing total Chol, LDL, TG, and malondialdehyde (MDA) concentrations, and increased HDL concentration and stimulated glutathione (GSH), SOD and CAT activities [79,80]. A recent in vitro study proved that both ethanolic and aqueous extracts of *T. spicata* aerial parts act as lipid lowering agents in a model of NAFLD by reducing the number of lipid droplets, the intracellular free radicals and lipid peroxidation [10] (Table 2).

Another species, *Thymbra capitata* (L.) Cav. (the synonym of *Thymus capitatus* (L.) Hoffmanns.), which is considered as a good ecological indicator of the dry Mediterranean area [122], is traditionally used in different European countries (Italy, Spain, and Portugal) for the treatment of cutaneous infections due to its powerful antiseptic properties [123]. Currently, the essential oil from *T. capitata* is greatly appreciated, especially in Portugal, and is endowed with several pharmacological properties such as antioxidant [124], anti-inflammatory [125], and anti-hyperglycemic activities by preventing lipid peroxidation, scavenging the peroxyl free radicals, and inhibiting lipoxygenase and α-amylase [125]. The essential oils of *T. capitatus* seem to improve liver damage in paracetamol-induced toxicity in rats by increasing SOD and GPx activities [81] (Table 2).

### 4.8. Orthosiphon aristatus (Blume) Miq.

*O. aristatus* (Blume) Miq. (synonym of *Orthosiphon stamineus*) is widely diffused in Southeast Asia regions such as Malaysia, Indonesia, and Thailand. It has been introduced as a culinary tea and was traditionally used for ameliorating rheumatism, hypertension, tonsillitis, epilepsy, menstrual disorders, gonorrhea, syphilis, renal calculus, hyperglycemia, and gallstones [126]. A phytochemical screening has identified the phenolic compounds isolated from this plant, including flavonol glycosides, lipophilic flavones, and caffeic acid derivatives (rosmarinic acid and 2,3-dicaffeoyltartaric acid) [127]. The first scientific studies have reported that methanolic and aqueous extracts from *O. aristatus* leaves have pharmacological activities such as anti-diabetic [128], hypolipidemic and anti-obesity [82] properties.

Using HFD-obese mice, Seyedan et al. observed that *O. aristatus* ethanolic extract could reduce body weight and also the serum TG, Chol, and LDL levels resulting in a significant reduction in fat accumulation, lipid peroxidation and stimulation of SOD activity in the liver [82]. Another study using diabetic rats showed that *O. aristatus*. aqueous extract possesses antihyperglycemic activity by boosting the expression level of Glucagon-like peptide 1 and ghrelin with the consequent reduction in glycemia and stimulation of insulin secretion [83] (Table 2).

### 4.9. Lycopus lucidus Turcz. ex Benth.

The genus *Lycopus* L. includes approximately 16 species, distributed in the Northern hemisphere and in the Eastern Asia. In Europe, we find the species *Lycopus europaeus* L. and *Lycopus exaltatus* L.f. The leaves are rich in flavonoids, coumarins, terpenoids, and tannins [129], and they have use in treating edema, wound healing, T2DM, and pain, especially in traditional Chinese medicine. *L. lucidus* has been suggested to play a protective role against obesity, NAFLD, and metabolic diseases. An in vitro study on HepG2 cells reported that the *L. lucidus* ethanolic extract significantly decreased the intracellular lipid accumulation in FFA-induced hepatic steatosis, and it was able to decrease the expression of lipogenic genes and increase β-oxidation [84]. An in vivo study showed that the administration of *L. lucidus* ethanolic extract to HFD mice significantly decreased body weight gain, serum ALT, LDL, Chol, GLU, insulin, leptin, and TNFα levels [84]. Moreover, both the liver weight and the hepatic TG and total Chol contents were significantly reduced, and these effects seem to be mediated by a down-regulation of sterol regulatory element-binding protein-1 (SREBP-1) and the activation of AMPK and PPARα. On the other hand, Lee et al. [85] observed that the aqueous extract of L. lucidus suppressed vascular inflammation in HUVEC exposed to high GLU through the attenuation of the crassulacean acid metabolism, and inhibited ROS production and suppressed the translocation and transcriptional activity of NFκB.

The hepatoprotective effect of a phenolic enriched extract from *L. lucidus* roots was also reported by an in vivo study using CCl_4_-induced hepatotoxicity [86]. In detail, the oral administration of this extract to mice significantly reduced the CCl_4_-induced increase of serum ALT, AST, ALP, TG, total Chol, and total bilirubin. The extract treatment also increased the hepatic GSH content and stimulated the activity of antioxidant enzymes SOD and CAT, decreased the hepatic MDA level, and prevented the deoxyribonucleic acid (DNA) fragmentation (Table 2).

### 4.10. Scutellaria baicalensis Georgi

*S. baicalensis* Georgi, also called Baikal Skullcap, is a perennial herb that is widely distributed in East Asia, including China, Japan, and Mongolia, where it is traditionally employed for treating inflammation, jaundice, and liver disorders [130]. Its dried roots are used in traditional Chinese medicine. Modern pharmacological studies reported that the bioactive compounds of *S. baicalensis* plays has many pharmacological effects, including anti-oxidant [131] and anti-inflammatory ones [89].

In both fatty acid-loaded hepatic HepG2 cells, an in vitro model of NAFLD, and in NAFLD mice and rats, a water extract from *S. baicalensis* roots ameliorated fat-induced lipotoxicity through the AMPK-mediated SREBP signaling pathway [87]. Moreover, the *S. baicalensis* water extracts seem to reduce weight gain, hypertriglyceridemia, and hyperinsulinemia, and restored metabolic process and insulin signaling pathways in obese mice [88]. *S. baicalensis* can help in improving insulin resistance by decreasing the levels of fasting and postprandial GLU, fasting insulin, homeostatic model assessment for insulin resistance, TGs, and LDL-Chol, and preventing inflammation by lowering the expression of inflammatory gene (TNF-α, IFN-γ, and F4/80) in HFD-induced insulin-resistant mice [89]. On the other hand, *S. baicalensis* methanol extract was able to prevent liver fibrosis and reduce the levels of liver hydroxyproline and lipid peroxidation induced by bile duct ligation and scission or CCl_4_ in rats [130] (Table 2).

### 4.11. Ocimum Species

*Ocimum* genus, a widely grown herb native to areas in Asia and Africa, Central and South America, includes approximately 150 species with well-known therapeutic properties. The most important are *O. gratissimum* L., *O. basilicum* L., and *O. tenuiflorum* L. [132].

*O. gratissimum* L. ameliorated the estrogen deficiency-induced obesity in ovariectomized rats mimicking menopausal women [90]. *O. gratissimum* water extract significantly reduced body weight gain and adipocyte size, suggesting that *O. gratissimum* L. dietary supplements may be useful in controlling the body weight of menopausal women.

*O. tenuiflorum* L. (the synonym of *Ocimum sanctum*) as a tea infusion seems to improve liver disease and lipid metabolism in diet-induced obese rats by reducing hepatic lipid accumulation through the down-regulation of lipogenesis and the up-regulation of mitochondrial fatty acid uptake, ameliorating insulin resistance and oxidative damage by stimulating the activity of the hepatic antioxidant enzymes glutathione s transferase (GST), GPx, and CAT [91]. *O. tenuiflorum* aqueous extracts from leaves exerted a lipid-lowering and antioxidant effect in rats fed with a high-Chol diet [92], where it decreased lipid accumulation in the liver and hyperlipidemia in blood, as well as enhanced liver antioxidant defence enzymes. Similar outcomes were found in *O. tenuiflorum* essential oils [94], which displayed anti-diabetic effects, by lowering the blood GLU, the serum lipid profile, and the serum creatinine effect in diabetic rats [93] (Table 2). On the other hand, *O. tenuiflorum* ethanolic extract in diabetic rats modulated glycogen and enzymes of carbohydrate metabolism such as Glucokinase (GK), hexokinase, and phosphofructokinase (PFK) [133].

Other plants of the *Ocimum* genus seem to have anti-diabetic potential in animal models. An anti-hyperlipidemic effect was reported for *O. basilicum* L. extract on rats [134]. In addition, *O. basilicum* extract showed anti-inflammatory activity on an in vitro model of obesity-induced inflammation [135]. Besides the anti-obesity and anti-diabetic effects, the antioxidant, anti-inflammatory, and anti-fibrosis effects of *Ocimum* spp. such as *O. gratissimum*, *O. basilicum*, and *O. tenuiflorum* have also been reported [136,137,138].

In humans, *O. tenuiflorum* consumption improved the serum total TG, total Chol, body mass index (BMI), plasma insulin, and insulin resistance in young overweight and obese subjects [139]. In NAFLD patients, *O. basilicum* seeds supplementation significantly reduced BMI [140].

### 4.12. Mesona chinensis Benth

*M. chinensis* Benth. is a perennial herbaceous plant widely distributed in Southeast Asia and China. Traditionally, it is consumed as an herbal drink and a gelatine dessert, but it has also been used for the treatment of heat-shock, hypertension, fever, inflammatory, T2DM and liver diseases [117]. Interests were focused on its polysaccharides, which include mainly galactose, GLU, rhamnose, arabinose, mannose, and uronic acid, as they possess multiple bioactivities, such as antioxidant activity, anti-diabetes activity, anti-hypertensionactivity, and the prevention of heat stroke [141].

An in vitro study reported a significant activity of *M. chinensis* aqueous extract against oxidative stress tested in terms of DPPH, superoxide and hydroxyl radical scavenging assays, the ferric reducing antioxidant power assay, oxygen radical absorbance capacity, and ferrous ion chelating activity [117]. In the same paper, the antioxidant and postprandial glycemia status after *M. chinensis* whole plant boiling water extract consumption in humans was studied, the results reported a decrease in postprandial plasma GLU, MDA, and serum TG levels, and an increase in plasma antioxidant capacity after the consumption of a high carbohydrate meal together with *M. chinensis* in 40 overweight participants of both genders and of different ages, suggesting that *M. chinensis* may have the potential for the prevention of chronic conditions and diseases associated with overweight and obesity [117]. Another study carried on mouse macrophage like cells (RAW) 264.7 cells and on cyclophosphamide-induced immune deficient mice showed an antioxidant activity of the polysaccharide extract from *M. chinensis* whole plant powder, which stimulated the antioxidant enzymes SOD, CAT and GPx compared to non-treated controls, while it also showed decreased levels of MDA, indicating less lipid peroxidation [95] (Table 2).

### 4.13. Lenotis leonurus (L.) R.Br.

*L. leonurus* is a shrub growing mainly in South Africa. The protective activities of chloroform, ethanol or acetone extracts from leaves and flowers of *L. leonurus* have been reported in several studies. The anti-inflammatory and anti-diabetic effects of a *L. leonurus* leaf extract were shown in two different rats’ models, where *L. leonurus* inhibited fresh egg albumin-induced paw edema, and played hypoglycemic effects in streptozotocin-induced diabetic rats [96]. Another study compared the effects of *L. leonurus* leaf organic extract with those played by murbiin, a diterpenoid labdane lactone abundant in *L. leonurus* leaves. To investigate and determine the mechanism of the hypoglycemic activity of this extract, the INS-1 rat cells cultured under hyperglycemic conditions or obese rats were treated with *L. leonurus*, the results showed increased insulin and GLUT2 gene expressions in INS-1 cells, and increase in respiratory rate and mitochondrial membrane potential. These extracts increased insulin secretion, HDL-Chol, restored total Chol, LDL-Chol, atherogenic index, IL-1β and IL-6 levels to their normal levels, suggesting the potential role of *L. leonurus* in the alleviation of diabetic symptoms [97]. Furthermore, the antidiabetic and anti-inflammatory effects of *L. leonurus* leaf extract were investigated in both cell models involving 3T3-L1 (fat), Chang (liver), C2C12 (muscle), and INS-1 (pancreatic) cells and in obese rats, thus proving that the mechanism of action is mainly at the adipose tissue level through increases in PPARγ, glucokinase, FAS and UCP2 gene expression [98] (Table 2).

## 5. Conclusions

The Lamiaceae family is one of the most important herbal families with a wide distribution in different natural ecosystems. It includes a wide variety of plants with a myriad of applications. Besides their culinary uses, members of the Lamiaceae family are employed in many industries, including cosmetics, fragrance, and perfumery. The most abundant genera belong to *Salvia*, *Scutellaria*, and *Stachys*, and the most known members are aromatic herbs.

Species of this family contain a lot of bioactive secondary metabolites and are extremely rich in (poly) phenols including phenolic acids, phenolic monoterpenoids, and flavonoids. Often Lamiaceae plants share a similar profile of (poly) phenolic compounds, i.e., carvacrol seems to be constantly present in a bundle of species [57,127]. As with most aromatic plants, essential oils are produced from many species of the Lamiaceae family, and many volatile substances are isolated from different plant parts (leaves, flowers, seeds, roots, and fruits) [1]. Essential oils from Lamiaceae are widely used in phytotherapy for their antimicrobial and antifungal [142], antidiabetic, and [19], immunoregulatory [20] properties.

**Figure 4:**
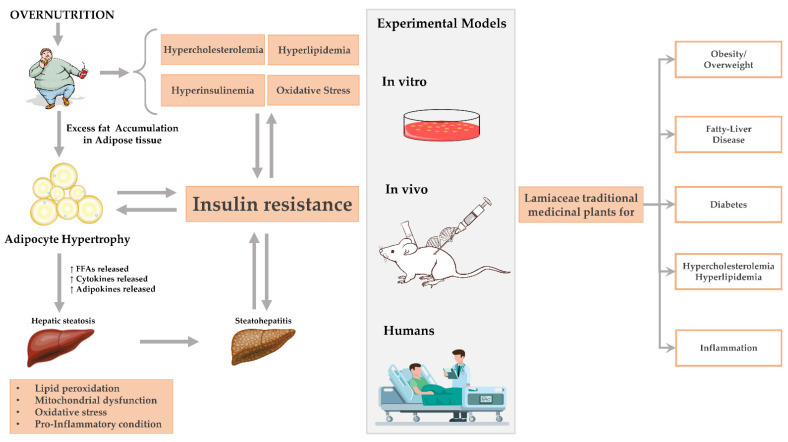
A schematic cartoon showing the main complications resulting from overnutrition and obesity: primarily liver steatosis and the mechanistic road for its progression, displaying also the potency of Lamiaceae plants in treating these complications in several experimental models (in vitro, in vivo, and clinical trials).

Globally, and mainly in Western countries, there is an increasing prevalence of obesity, NAFLD and other obesity-related diseases as a consequence of overnutrition [143]. In the absence of a definitive medical treatment so far, the use of bioactive plant-based natural products is considered a safety approach. Among the numerous natural sources, Lamiaceae plants seem to be promising for treating and/or preventing the development of hepatic steatosis. This aspect is directly related to the presence in its parts of a variety of phenolic compounds that made this family one of the best candidates for treating several illnesses since ancient times. In particular, both in vitro and in vivo studies (Table 1), as well as clinical trials (Table 2), demonstrated the anti-hyperlipidemic, anti-diabetic, and hypoglycemic properties of *S. officinalis*, *S. hispanica*, *R. officinalis*, *M. spicata*, *T. vulgaris*, and *S. baicalensis*. Indeed, Figure 4 shows the different complications that resulted from overnutrition, including obesity-related disorders; mainly liver steatosis and the mechanistic road for its progression, displaying the potency of Lamiaceae plants in treating these complications in several experimental models: in vitro, in vivo, and in clinical trials.

In this review, we have summarized the most important in vitro, in vivo, and human studies about the potential of a selected group of Lamiaceae plants in treating or alleviating metabolic disorders acting as anti-obesity, anti-diabetic, anti-inflammatory, and anti-oxidative agents. The list of analyzed plants in this review, including 13 species, is briefly summarized concerning their applications (Table 3) that might be attributed to the bioactive and healthy compounds abundantly found in these plants, which are structurally presented in Figure 2. Carvacrol, which is presented in *Thymbra* and *Thymus* species, and rosmarinic acid, which is highly abundant in *Orthosiphon aristatus*, *Lycopus lucidus*, and *Melissa officinalis*, are the most diffused compounds among the selected group of plants. Also, flavonoids as apigenin and quercetin are very present in *Ocimum* spp, *Salvia hispanica*, and *Leonurus sibiricus*. Based on the abundance of polyphenols, these plants showed great potential in treating obesity and metabolic-related disorders.

Interestingly, the role of natural products on drug development has been increasing, not only when the bioactive compounds are directly used as therapeutic agents but also when they are used as raw material for drug synthesis, or as a base model for new biologically active compounds. In this context, several innovative drugs are compositionally based on one or more of the Lamiaceae plants as therapeutic resources in the form of herbal infusion, pharmaceutical preparations such as extracts, tablets or capsules by extracting and purifying active compounds. To give examples, *R. officinalis*, *T. vulgaris*, *O. sanctum*, *M. officinalis*, and *O. vulgare* are widely used in the industry of drugs targeting cardiovascular diseases, atherosclerosis, and lipid peroxidation.

As a final consideration, the use of these plants as a nutraceutical intervention targeting obesity and related metabolic syndrome could be an effective strategy to mitigate the obesity epidemic. Overall, although there is promising evidence of the efficacy of the Lamiaceae genus in the treatment of metabolic-associated disorders, the data are too preliminary and mostly fail to explain the exact cellular and molecular mechanisms of action and the respective active compounds. Moreover, the effectiveness of these medicinal herbal plants is not certain due to some limitations, such as the small sample size and the short duration of studies. Therefore, further preclinical and clinical studies are essential, with a larger sample size and a more structured methodology in order to investigate the mechanisms of actions, realistic dosages, clinical efficacy, and safety of the extracts and active compounds in treatment. This deeper investigation provides a great opportunity to rely exclusively on plants and their extracts in the drug discovery and development for the treatment of several chronic diseases. This review provides a useful approach for the further identification of new compounds from various medicinal plants which may be effective in disease treatment.
